# External validation of a deep learning electrocardiogram algorithm to detect ventricular dysfunction

**DOI:** 10.1016/j.ijcard.2020.12.065

**Published:** 2021-04-15

**Authors:** Itzhak Zachi Attia, Andrew S. Tseng, Ernest Diez Benavente, Jose R. Medina-Inojosa, Taane G. Clark, Sofia Malyutina, Suraj Kapa, Henrik Schirmer, Alexander V. Kudryavtsev, Peter A. Noseworthy, Rickey E. Carter, Andrew Ryabikov, Pablo Perel, Paul A. Friedman, David A. Leon, Francisco Lopez-Jimenez

**Affiliations:** a Department of Cardiovascular Medicine, Mayo Clinic, Rochester, MN, USA; b Department of Infection Biology, London School of Hygiene and Tropical Medicine, London WC1E 7HT, UK; c Department of Health Sciences Research, Mayo Clinic, Jacksonville, FL, USA; d Department of Infectious Disease Epidemiology, London School of Hygiene & Tropical Medicine, London WC1E 7HT, UK; e Northern State Medical University, Arkhangelsk 163000, Russia; f Department of Community Medicine, UiT The Arctic University of Norway, Tromsø 9037, Norway; g Institute for Clinical Medicine, University of Oslo, Campus Ahus, Lørenskog PB 1000 1478, Norway; h Department of Cardiology, Akershus University Hospital, 1478 Nordbyhagen, Oslo, Norway.; i Novosibirsk State Medical University, Russian Ministry of Health, Novosibirsk 630091, Russia; j Department of Non-communicable Diseases Epidemiology, Faculty of Epidemiology and Population Health, London School of Hygiene & Tropical Medicine, London WC1E 7HT, UK; k International Laboratory for Population and Health, National Research University, Higher School of Economics, Moscow, Russia; l Research Institute of Internal and Preventive Medicine, Branch of Institute of Cytology and Genetics, Siberian Branch of the Russion Academy of Sciences, Novosibirsk 630090, Russia; m Department of Experimental Cardiology, University Medical Center Utrecht, Netherlands

**Keywords:** AI-ECG, artificial-intelligence electrocardiogram algorithm, AUC, area under the curve, CNN, convoluted neural network, LVEF, left ventricular ejection fraction, LVSD, left ventricular systolic dysfunction, NPV, negative predictive value, PPV, positive predictive value, TTE, transthoracic echocardiography, Artificial intelligence, Machine learning, Electrocardiogram, Left ventricular systolic dysfunction

## Abstract

**Objective:**

To validate a novel artificial-intelligence electrocardiogram algorithm (AI-ECG) to detect left ventricular systolic dysfunction (LVSD) in an external population.

**Background:**

LVSD, even when asymptomatic, confers increased morbidity and mortality. We recently derived AI-ECG to detect LVSD using ECGs based on a large sample of patients treated at the Mayo Clinic.

**Methods:**

We performed an external validation study with subjects from the Know Your Heart Study, a cross-sectional study of adults aged 35–69 years residing in two cities in Russia, who had undergone both ECG and transthoracic echocardiography. LVSD was defined as left ventricular ejection fraction ≤ 35%. We assessed the performance of the AI-ECG to identify LVSD in this distinct patient population.

**Results:**

Among 4277 subjects in this external population-based validation study, 0.6% had LVSD (compared to 7.8% of the original clinical derivation study). The overall performance of the AI-ECG to detect LVSD was robust with an area under the receiver operating curve of 0.82. When using the LVSD probability cut-off of 0.256 from the original derivation study, the sensitivity, specificity, and accuracy in this population were 26.9%, 97.4%, 97.0%, respectively. Other probability cut-offs were analysed for different sensitivity values.

**Conclusions:**

The AI-ECG detected LVSD with robust test performance in a population that was very different from that used to develop the algorithm. Population-specific cut-offs may be necessary for clinical implementation. Differences in population characteristics, ECG and echocardiographic data quality may affect test performance.

## 1 Introduction

Left ventricular systolic dysfunction (LVSD), even when asymptomatic, is associated with increased cardiovascular morbidity and mortality [1]. There is currently no cost-effective method to screen for LVSD, and routine echocardiography in the general population is not recommended [2]. Yet, given its association with increased morbidity and mortality and the availability of effective treatments to prevent progression, there is likely to be clinical benefit in identifying subjects with asymptomatic LVSD. In low income countries, where routine echocardiography may be too costly, a simple and inexpensive screening test for those with symptoms suggestive of undiagnosed heart failure can be lifesaving.

Recently, we developed an artificial intelligence electrocardiogram (AI-ECG) algorithm to detect LVSD, defined as left ventricular ejection fraction (LVEF) less than or equal to 35%. By analysing paired ECG and transthoracic echocardiography (TTE) data in 44,959 subjects treated at Mayo Clinic, we trained a convolutional neural network (CNN) to identify subjects with LVSD with a sensitivity and specificity of 86.3% and 85.7% respectively (AUC of 0.93) in an independent retrospective internal validation study [3]. We further performed a prospective validation of the ECG algorithm on an internal study of 8600 prospective subjects and demonstrated similar model performance [4].

Yet, even with this encouraging data, in order to be able to generalize our findings, it is imperative to evaluate how well these algorithms work in other populations, in different clinical settings, and with variable ECG and echocardiographic data collection techniques. There have been several well publicized examples of initially promising algorithm that failed to generalize to broader populations. Notable examples include poor performance of facial recognition software in diverse populations and difficulty generalizing retinal image diagnostics [5]. Such problems can arise when datasets used to train the algorithms are of limited diversity. Additionally, if there is excessive data redundancy in internal validation, these algorithms may have a tendency to “overfit” to a given dataset [6].

To evaluate the robustness and accuracy of our algorithm, we performed an external validation of our AI-ECG to detect LVSD in a completely different setting and population from a population-based cross-sectional study of men and women living in two Russian cities.

## 2 Methods

### 2.1 Study design

We performed the retrospective external validation study of our deep learning ECG algorithm to detect LVSD (defined as LVEF ≤ 35%) in a study of subjects from the cross-sectional Know Your Heart Study in Russia [7]. The primary outcome was the predictive ability of the deep learning network to identify subjects with LVSD from ECGs from this external validation study. The study was approved by the Mayo Clinic Institutional Review Board and by the ethics committees of the London School of Hygiene & Tropical Medicine, Novosibirsk State Medical University, the Institute of Preventative Medicine, Novosibirsk and the Northern State Medical University, Arkhangelsk.

Twelve‑lead ECGs were obtained using Cardiax devices (IMED Ltd., Hungary) with a sampling rate of 500 MHz, and were further filtered to 0.1 MHz to 100 MHz to eliminate noise and artefact. ECGs shorter than 10 s were excluded (three subjects). ECGs longer than 10 s were truncated to 10 s. Of note, the algorithm was previously trained on 10 s ECGs raw signals sampled at 500 MHz as highlighted below. TTE was performed using GE VividQ machines (GE Healthcare) in accordance with a strict-defined protocol [7]. Two methods of LVEF were used in the Know Your Heart Study (biplane and Teichholz formula). For LVEF determination, initial comparative analysis of the two LVEF calculations revealed poor correlation between the two calculation methods (R-squared 0.3). Therefore, as the biplane method is generally considered more accurate than the Teichholz formula, only subjects with LVEF determined by the biplane method were used for the final analysis.

### 2.2 Participants

We included participants from the Know Your Heart Study, a large cross-sectional population-based study of adults aged 35 to 69 year in two Russian cities, Arkhangelsk and Novosibirsk. Extensive information on socioeconomic status, laboratory studies, ECGs and TTEs were collected on all subjects as part of the study. Subjects had comprehensive cardiovascular screening performed, including ECG and TTE. The study protocol for the Know Your Heart Study is published and described in detail [7].

### 2.3 Test methods and the convolutional neural network

The convolutional neural network (CNN) based algorithm has been previously described, developed and internally validated [3]. The network was trained with Keras with a Tensorflow (Google, Mountain View, CA) backend. No additional training or optimization was performed on this sample. The sole input for the model was a 12‑lead ECG (10 s duration sampled at 500 Hz) divided into two second segments with an overlap of one second. The network included six single‑lead convolutional layers followed by a nonlinear “Relu” activation function, a batch normalization layer and a maximum pooling layer. These were then fused in another convolutional layer with simultaneous access to all leads. This data was the input for a fully connected network with two hidden layers with dropout layers to reduce overfitting and an output layer that was activated using the “Softmax” function. The outputs of the softmax layer were compared with the binary LVSD label (LVEF ≤ 35% or LVEF > 35%). With the Adam optimizer, the layers were trained to classify each ECG into one of these two groups [8]. The output for the network is a continuous value between 0 and 1 representing the probability of LVSD.

For the external validation study, the ECGs (10 s duration at variable sample rates) were fed into the pre-existing network utilizing the same probability threshold for a positive screen (0.256) as determined by the original interval validation study. Using the cut-off value, all tests either had a positive or negative screen. None of the tests were considered indeterminate.

### 2.4 Analysis

For the primary objective of evaluating the AI-ECG performance, a comprehensive panel of diagnostic performance metrics were summarized. In particular, the accuracy, sensitivity, specificity, negative predictive value (NPV), positive predictive value (PPV), and area under the receiver operating curve (AUC) of the validation study were determined using the original model output cut-off of greater than or equal to 0.256, indicating that the input ECG had at least a 25.6% probability of LVSD [3]. 95% confidence intervals were used to summarize sample variability in the estimates. Alternative study-specific cut-offs were explored based on clinical relevance. We examined the optimal threshold defined as the threshold that maximized the sum of sensitivity and specificity (i.e., Youden's index) along with thresholds that would provide 70%, 80% and 90% sensitivity. Given there was no external sample to validate these thresholds on, 1000 bootstrap replicates were generated using the R package cutpointR (version 1.0.0) in order to estimate the 95% bootstrap confidence interval for the candidate threshold. Continuous data are presented as mean ± SD and median and interquartile range (IQR) if highly skewed. Statistical analysis was performed using R (version 3.4.2).

## 3 Results

### 3.1 Study population

**Fig. 1:**
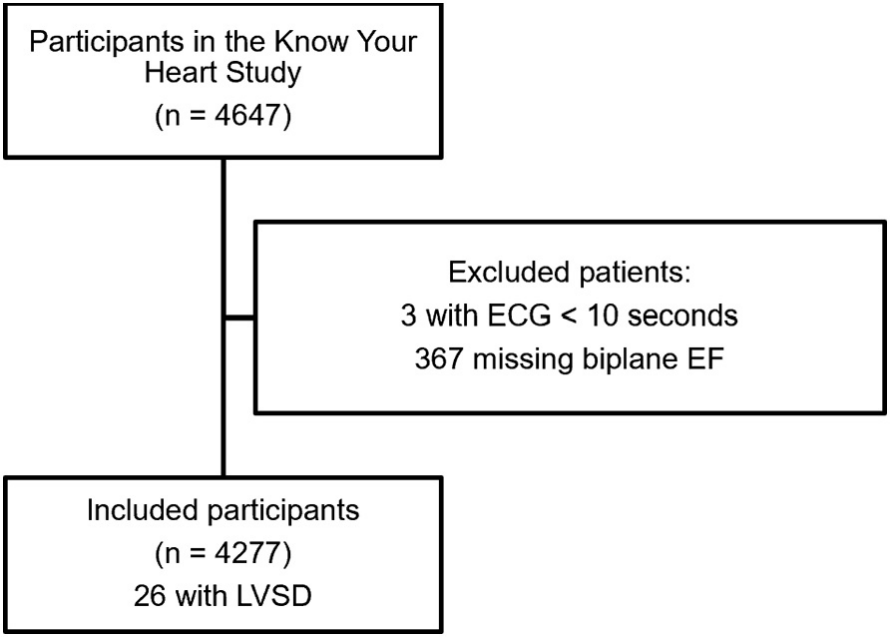
Derivation of included study participants.

Of the 4647 subjects in the Know Your Heart Study, 4277 (91.4%) subjects were included in the validation study, after exclusion of subjects with ECGs shorter than 3 s and missing biplane LVEF. The derivation of the final study population is shown in the flow diagram as Fig. 1.

Of these subjects, 56% were female with a mean age of 54.1 ± 9.7. When compared to the testing set in the original publication, the subjects in this external validation study were younger with fewer co-morbidities as was anticipated as the former was a clinical population while the latter is a random population sample of a general population of two Russian cities. There was a significantly lower proportion of subjects in the validation study with an existing self-reported diagnosis of heart failure. The difference in mean LVEF was not clinically significant. The data is summarized in Table 1 and the Supplemental Data. Notably, only 26 subjects (0.6%) had LVSD in the present study, compared to 7.8% with LVSD in the original study comprised of a clinical population undergoing diagnostic investigation.

**Table 1:** Patient self-reported demographic and comorbidity data.

	Know Your Heart validation set (*n*=4277)
Mean Age, years (SD)	54.1 (9.7)
Female, n (%)	2403 (56)
Mean Ejection fraction (SD)	55.8 (6.1)
Heart failure, n (%)	569 (14)
Diabetes mellitus, n (%)	344 (8)
Hypertension, n (%)	2052 (48)
Myocardial infarction, n (%)	245 (6)

### 3.2 Outcomes

#### 3.2.1 Diagnostic performance in the Know Your Heart study

**Fig. 2:**
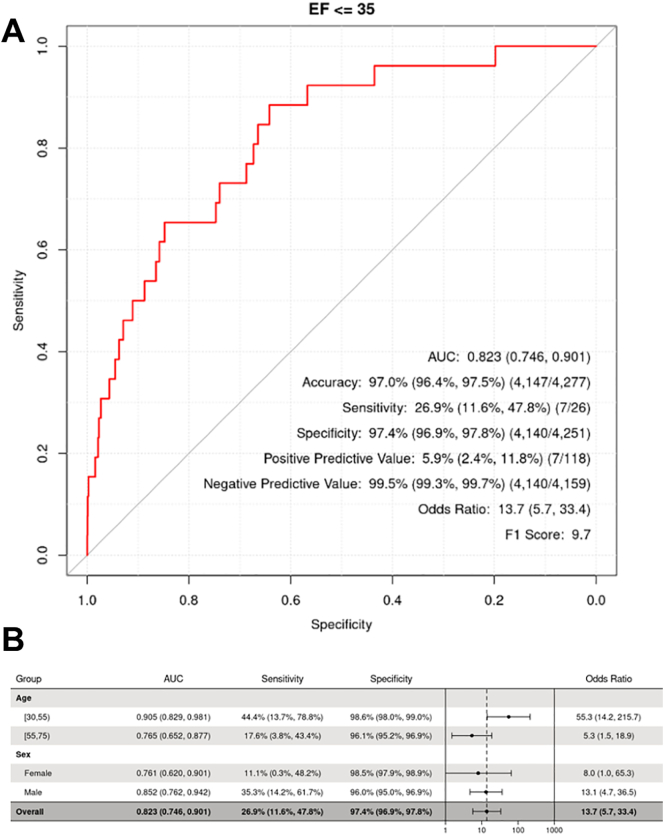
Test performance of AI-ECG, overall and subgroups by age and sex. (A) ROC of AI-ECG with test performance characteristics using an LVSD probability cut-off of 0.256. (B) Test performance in subgroups by age and sex using an LVSD probability cut-off of 0.256.

Of the 4277 unique ECGs processed into the CNN algorithm, 118 subjects were identified as having LVSD using the pre-existing probability cut-off of 0.256 used in the previous studies. The AUC showed very good discrimination (0.82, 95% CI: 0.75–0.90) Sensitivity, specificity, and accuracy were 26.9% (95% CI: 11.6–47.8%), 97.4% (95% CI: 96.9–97.8%), and 97.0% (95% CI, 96.4–97.5%), respectively. Using the original cut-off, the PPV of a positive screen was 5.9%, whereas the NPV was 99.5%. The receiver operating characteristic curve is shown in Fig. 2a.

We further evaluated the test performance using different cut-off values of LVSD probability. To maximize the sum of sensitivity and specificity, a cut-off value of 0.0163 (95%CI: 0.013–0.040) was identified from the data. With this cut-off, 1543 (36%) of subjects screened positive, with a sensitivity, specificity, PPV, NPV and accuracy of 84.6%, 64.2%, 1.4%, 99.9%, and 64.3%, respectively. Cut-off values to explore sensitivities of 70%, 80%, and 90% were also determined, and the test performance parameters are shown in Table 2. Subgroup analyses for age (greater than or less than the mean age of 55) and sex were also performed and summarized in Fig. 2b.

**Table 2:** Test performance of AI-ECG using different LVSD probability cut-off values.

LVSD probability cut-off value	Positive tests	Sensitivity	Specificity	PPV	NPV	Accuracy
0.256	118 (3%)	26.9%	97.4%	5.9%	99.5%	97.0%
0.0163	1543 (36%)	84.6%	64.2%	1.4%	99.9%	64.3%
0.0127	1866 (44%)	92.3%	56.7%	1.3%	99.9%	56.9%
0.0180	1413 (33%)	80.8%	67.3%	1.5%	99.8%	67.3%
0.0239	1131 (26%)	73.1%	73.8%	1.7%	99.8%	73.8%

Abbreviations: PPV, positive predictive value; NPV, negative predictive value.

Given the significantly lower prevalence of LVSD in the present study, we modelled the PPVs and NPVs using the fixed sensitivity (84.6%) and specificity (64.2%) from our maximized cut-off value of 0.0163 under different theoretical prevalence values of LVSD in the population. As expected, the NPV and PPV increase with increasing disease prevalence. The results are shown in Table 3.

**Table 3:** Impact of disease prevalence on positive predictive value (PPV), negative predictive value (NPV) and accuracy using the maximized LVSD probability cut-off of 0.0163.

Disease prevalence	PPV	NPV	Accuracy
0.6%	1.4%	99.9%	64.3%
2%	4.6%	99.5%	64.6%
4%	9.0%	99.0%	65.0%
6%	13.1%	98.5%	65.4%
8%	17.0%	98.0%	65.8%
10%	20.8%	97.4%	66.2%
20%	37.1%	94.3%	68.3%

Abbreviations: PPV, positive predictive value; NPV, negative predictive value.

## 4 Discussion

We performed an external validation study of the AI-ECG algorithm in a study of 4277 adult Russian subjects who were part of the cross-sectional population-based Know Your Heart Study. We found that applying the algorithm to this study using the original model cut-off yielded a very good discrimination (AUC of 0.82), high specificity and accuracy, but low sensitivity, for detecting LVSD. When the probability threshold of LVSD was lowered from 0.256 to the study-specific maximized threshold, the test performance characteristics were improved for this study population. These findings suggest that the AI-ECG algorithm has clinically robust performance in a clinically distinct population sample, but that population-specific cut-offs may be necessary to optimize test performance for populations with different characteristics including different underlying disease prevalence.

When we compare this external validation study to the previous internal validation studies performed, there are differences in the performance characteristics of the algorithm in the external study. Compared to both the original validation study as well as the second prospective internal validation study, application of the algorithm to the present external validation study yields a modestly lower accuracy, sensitivity and specificity, even when evaluating the model performance at the study-specific optimal LVSD probability threshold of 0.0163 [3,4]. Likewise, we report an AUC of 0.82 with the external study, compared to 0.93 of the original internal validation study and 0.92 of a second internal validation study, which while lower still represents a good measure of separability. It is unclear whether the modest degradation in performance compared with our initial publications stems from the ECG signal processing adjustments required or from variations in the echocardiographic assessment of the LVEF (the gold standard in this case). However, in our sensitivity analyses, actual and modelled variation of EF measurement method by TTE and variations in ECG acquisition duration did not appear to significantly affect test performance. Furthermore, our AI-ECG was developed using subjects seen in a clinical setting, where subjects presumably have more co-morbidities and previous events that warranted ECG and TTE. This contrasts with the present study population, which is population-based.

We explored study-specific cut-offs to evaluate different clinical screening scenarios. To achieve high (90%) sensitivity, the cut-off was lowered, resulting in nearly half of the subjects screening positive, with low PPV and accuracy. While a screening test should ideally have high sensitivity, the cost of screening in such a manner may be cost-prohibitive if nearly half of patients who screen positive require further expensive testing with a yield of just over 1%. Therefore, as with any test, it is important to evaluate clinical goals as well as pragmatic cost concerns. When we evaluated a lowered sensitivity by increasing the test cut-off, naturally, less patients screen positive and the PPV and accuracy improves, while maintaining a high NPV, given the low prevalence of LVSD. The test performance of the AI-ECG algorithm may be further augmented by other historical, clinical, laboratory and radiographical features that modulate pre-test probability [4].

Previous non-AI models and scoring systems had been developed to utilize ECGs to screen for LVSD, though none are widely used in clinical practice. One study in 10,782 patients found that certain ECG findings, such as wide QRS, prolonged QT, delayed transition, LVH, AV block, have been associated with LVSD. Six key ECG findings were used to create an unweighted score. A score of > 3 had a sensitivity of 0.443, specificity of 0.947, PPV of 0.326, and NPV of 0.961, demonstrating high specificity but low sensitivity with a cohort LVSD prevalence of 12.4% [9]. Another study of 14,507 patients at the Mayo Clinic showed that certain ECG characteristics including T-wave inversions, left bundle branch block, LVH, evidence of myocardial infarction were significant predictors of LVSD. When combined with other clinical variables such as cardiomegaly on chest X-ray, basilar rales on examination and third heart sound, a logistic regression model was developed with a sensitivity of 0.68 and specificity of 0.74 [10]. Overall, while direct comparisons between the various non-AI models are not possible, the AI-ECG algorithm appears to be more sensitive with robust test performance.

Furthermore, the algorithm performance remains with a high AUC, and the sensitivity and specificity are comparable to routine testing used currently in cardiovascular medicine (stress echocardiography, nuclear perfusion imaging) [11]. There is a paucity of data in regards to asymptomatic LVSD, and no guidelines currently recommend routine screening given lower-yield and high-cost of imaging. Yet, there is growing evidence that screening higher-risk groups (i.e. higher pre-test probability), such as older subjects with hypertension, diabetes, greater body mass index, etc. may have clinical utility, given the morbidity and mortality of incident congestive heart failure [12]. The ability to screen for asymptomatic LVSD using a simple and inexpensive modality, such as an ECG, can lead to early detection and treatment with medications and ultimately prevent the progression to clinical heart failure.

Certainly, the potential use of artificial intelligence to assist clinicians in daily practice and to advance the science of medicine is promising. But as with any algorithm to be clinically useful and scientifically sound, vigorous testing and validation must be performed in diverse clinical settings and populations – even beyond this external validation study. We have also shown that different populations may require different thresholds, and implementation may require initial calibration from population-based validation studies. In populations with known low prevalence of LVSD, the probability cut-off may need to be lowered in order to improve the sensitivity (i.e. decrease false negatives) of the AI-ECG as a potential screening tool, though at the expense of increasing false positives. Alternatively, such algorithms may need to be separately trained on data in different populations in order to maximize test performance. From a public health perspective, further investigation can be performed to evaluate the potential cost-effectiveness and impact on cardiovascular outcomes of a screening approach using this algorithm, especially in conjunction with other clinical and laboratory factors to improve predictive ability.

There are several important issues to be considered in interpreting the findings from this study. First, despite the acquisition of ECG data from hardware with filtering characteristics different than the hardware used to develop the algorithm, we show for the first time the ability to adjust and adapt the acquisition modalities to obtain clinically useful information. Given the broad array of potential sources of ECG data, input agility will be important for global utility to enable this massively scalable means of screening for an important disease. Indeed, we have previously shown the ability to use a smartphone-recorded ECG to determine blood potassium levels and have also demonstrated the ability to assess EF from a single lead, potentially making this tool available from a watch or phone [13]. Second, the incidence of heart failure is increasing throughout the world, and there remains a significant disparity in healthcare resources between countries [14]. Due to the fact that they are inexpensive to deploy and massively scalable, digital screening from 12 lead ECGs or ultimately smartphone-based ECGs may democratize access to care, and may provide critical tools to rural, resource-constrained environments to save lives.

### 4.1 Limitations

There were limitations in this study. Firstly, it should be noted that there were very few subjects in this external validation study with LVSD compared to the original study (0.6% versus 7.8%). This may partly reflect differences in the burden of different types of cardiovascular disease in the two populations. However, the most important explanation for this difference in prevalence is the fact that the population on which the algorithm was developed was a clinically, symptomatic population, while the validation study was based on a general population sample selected at random regardless of symptoms or health status. This may have impacted the overall predictive ability of the algorithm. In particular, as others have suggested, the sensitivity of a diagnostic test may be reduced in a low disease prevalence population because the cases may be less severe than in a population with high prevalence [15,16]. This would certainly be the case in our situation where we are effectively comparing a symptomatic clinical population with a general population sample.

Secondly, as the only input into the algorithm is the ECG data, differences in ECG quality may impact the quality of the predictions made by the algorithm. Lastly, there may be significant technical variations in the echocardiography between the studies. From initial analysis of the measurement of LVEF in the present study, we noted poor correlation and significant variability between two methods used to calculate LVEF. As we are using TTE as the gold standard in determining a positive or negative screen, the quality and reliability of the TTE is also of significant importance. It is possible that there may have been misclassification of subjects regarding actual LVSD status. To mitigate this, we included only patients with LVEF determined by the biplane method.

## Clinical perspectives

We performed an external validation study of an AI-ECG algorithm to detect LVSD in a clinically distinct population. While the test performance was modestly weaker between these different populations, the algorithm remains robust to detect LVSD and has comparable sensitivity, specificity and AUC to many commonly used cardiovascular medicine testing.

## Translational outlook

AI-ECG represents a readily scalable method of screening for LVSD, which can have immense impact in global health and public health, but rigorous calibration of the AI ECG to the population of interest and control of ECG and echocardiographic data quality is necessary. Furthermore, validation with other populations and clinical scenarios as well as investigations into cost-effectiveness and impact on clinical outcomes should be performed.

## Funding

The Know Your Heart study is a component of the International Project on Cardiovascular Disease in Russia (IPCDR). IPCDR was funded by a 10.13039/100010269Wellcome Trust Strategic Award (100217), supported by funds from the Norwegian 10.13039/501100003506Ministry of Health and Care Services, Norwegian Institute of Public Health, and 10.13039/100007465UiT The Arctic University of Norway. The funders had no role in study design, data collection and analysis, decision to publish, or preparation of the manuscript. DAL’s contribution was partly undertaken within the framework of the HSE University Basic Research Program.

## Contributorship statement

AST planned, conducted the study and wrote the manuscript. IZA planned and conducted the study and edited the manuscript. JMI and REC performed data analysis. EDB, TGC, SM, SK, HS, AVK, PAN, AR, PP, PAF and DAL edited the manuscript and contributed to its writing. FLJ planned the study, edited the manuscript and is the guarantor of the content.

## Declaration of Helsinki

The study complies with the Declaration of Helsinki. The study was approved by the Mayo Clinic Institutional Review Board and by the ethics committees of the London School of Hygiene & Tropical Medicine, Novosibirsk State Medical University, the Institute of Preventative Medicine, Novosibirsk and the Northern State Medical University, Arkhangelsk.

## Patient and Public Involvement Statement

This research was done without patient involvement. Patients were not invited to comment on the study design and were not consulted to develop patient relevant outcomes or interpret the results. Patients were not invited to contribute to the writing or editing of this document for readability or accuracy.

## Author statement

Itzhak Zachi Attia, M.S., PhD, conceptualization, methodology, editing.

Andrew S. Tseng, M.D., co-first author, conceptualization, analysis, writing, editing.

Ernest Diez Benavente, PhD, conceptualization, investigation, editing.

Jose R. MedinaInojosa, M.Sc, M.D., methodology, analysis.

Taane G. Clark, D.Phil, investigation, review and editing.

Sofia Malyutina, M.D., PhD Investigation, data acquisition, review and editing.

Suraj Kapa, M.D., investigation, review and editing.

Henrik Schirmer, M.D., PhD, investigation, review and editing.

Alexander V. Kudryavtsev, PhD, investigation, review and editing.

Peter A. Noseworthy, M.D., investigation, review and editing.

Rickey E. Carter, Ph.D., analysis, review and editing.

Andrew Ryabikov, M.D., investigation, review and editing.

Pablo Perel, M.D., investigation, review and editing.

Paul A. Friedman, M.D., resources, supervision, review and editing.

David A. Leon, PhD^.^, conceptualization, funding acquisition, editing.

Francisco Lopez-Jimenez, M.D., M.B.A., conceptualization, funding acquisition, investigation, methodology, project administration, supervision, review and editing.

## Declaration of Competing Interest

Mayo Clinic has licensed the underlying technology to EKO, a maker of digital stethoscopes with embedded ECG electrodes. Mayo Clinic may receive financial benefit from the use of this technology, but at no point will Mayo Clinic benefit financially from its use for the care of subjects at Mayo Clinic. P.A.F., F.L.-J., S.K., and Z.I.A. may also receive financial benefit from this agreement.

## Appendix A Supplementary data

Supplementary material Image 1
