# Supplementary material for: External validation of a deep learning electrocardiogram algorithm to detect ventricular dysfunction
Source: Int J Cardiol. 2021 Apr 15;329:130–5. doi: 10.1016/j.ijcard.2020.12.065 (PMC7955278; doi:10.1016/j.ijcard.2020.12.065)
Supplement: Supplementary file 1 — Supplementary material [file mmc1.docx]

SUPPLEMENTAL MATERIAL

***Supplemental Table 1:***

|  | Know Your Heart validation set (n=4,657) | Original test set (n=52870) | p-value |
| --- | --- | --- | --- |
| Age, years (SD) | 54.3 (9.7) | 61.8 (16.5) | <0.0001 |
| Female, n (%) | 2604 (56) | 22,704 (43) | <0.0001 |
| Mean EF (SD) | 55.8 (6.1) | 56.2 (12.0) | 0.03 |
| Heart failure, n (%) | 614 (13) | 1,803 (20) | <0.0001 |
| Diabetes mellitus, n (%) | 384 (8) | 12,433 (24) | <0.0001 |
| Hypertension, n (%) | 2259 (49) | 24,643 (47) | <0.0001 |
| Myocardial infarction, n (%) | 270 (6) | 6,770 (13) | <0.0001 |

***Supplemental Methods and Results for Sensitivity Analyses***

We performed sensitivity analyses to evaluate the impact of echocardiographic data variability and quality on the performance of the deep-learning algorithm. First, we performed an analysis excluding the first 200 TTEs performed at each center to account for inaccuracies arising from initial inexperience. The AUC was 0.85 with a sensitivity and specificity of specificity of 74.1% and 84.4%, respectively.

We then evaluated the absolute differences in EF measurement by the Teichholz and biplane methods. When excluding subjects with differences in EF of greater than 10%, the AUC was 0.83 with a sensitivity and specificity of 63.0% and 84.7%, respectively. When we exclude subjects with differences in EF of greater than 1 standard deviation from the mean (4.5%), the AUC was 0.84, with a sensitivity and specificity of 70.8% and 84.6%, respectively.

Given the variability and ECG duration, we performed a sensitivity analysis limiting ECG data to the first 10 seconds of an ECG. The AI-ECG was applied to the first 10 seconds of each ECG with a resultant test performance similar to that of the main model with an AUC of 0.83.

In regards to sample population, 254 subjects within the Know Your Heart study were part of the Narco study. These patients had high rates of alcoholism, not necessarily reflective of the general population. When these patients were excluded, the resultant test performance was similar to that of the main model with an AUC of 0.83.
